# The Significance of Bile in the Biliopancreatic Limb on Metabolic Improvement After Duodenal-Jejunal Bypass

**DOI:** 10.1007/s11695-024-07176-7

**Published:** 2024-03-21

**Authors:** Tomomi Kawana, Hirofumi Imoto, Naoki Tanaka, Takahiro Tsuchiya, Akihiro Yamamura, Fumito Saijo, Masamitsu Maekawa, Toru Tamahara, Ritsuko Shimizu, Kei Nakagawa, Shinobu Ohnuma, Takashi Kamei, Michiaki Unno

**Affiliations:** 1 Department of Surgery, Tohoku University Graduate School of Medicine, 1-1, Seiryo-Machi, Aoba-Ku, Sendai, 980-8574 Japan; 2 Department of Pharmaceutical Sciences, Tohoku University Hospital, Sendai, Japan; 3 Tohoku University, Tohoku Medical Megabank Organization, Sendai, Japan

**Keywords:** Duodenal-jejunal Bypass, Bile acids, Enterohepatic circulation

## Abstract

**Introduction:**

Duodenal-jejunal bypass (DJB) is an experimental procedure in metabolic surgery that does not have a restrictive component. Changes in bile acid (BA) dynamics and intestinal microbiota are possibly related to metabolic improvement after DJB. Our previous studies involving obese diabetic rats showed the crucial role of the biliopancreatic limb (BPL) in metabolic improvement after DJB caused by BA reabsorption. We established a new DJB procedure to prevent bile from flowing into the BPL and aimed to elucidate the importance of bile in the BPL after DJB.

**Methods:**

Otsuka Long-Evans Tokushima Fatty rats with diabetes were divided into three groups: two DJB groups and a sham group (*n* = 11). Duodenal-jejunal anastomosis was performed proximal to the papilla of Vater in the DJB group (*n* = 11). However, the DJB-D group (*n* = 11) underwent a new procedure with duodenal-jejunal anastomosis distal to the papilla of Vater for preventing bile flow into the BPL.

**Results:**

Glucose metabolism improved and weight gain was suppressed in the DJB group, but not in the DJB-D and sham groups. Serum BA level and conjugated BA concentration were elevated in the DJB group. The gut microbiota was altered only in the DJB group; the abundance of *Firmicutes* and *Bacteroidetes* decreased and that of *Actinobacteria* increased. However, the DJB-D group exhibited no apparent change in the gut microbiota, similar to the sham group.

**Conclusion:**

BAs are essential in the BPL for metabolic improvement after DJB; they can improve the gut microbiota in these processes.

**Graphical Abstract:**

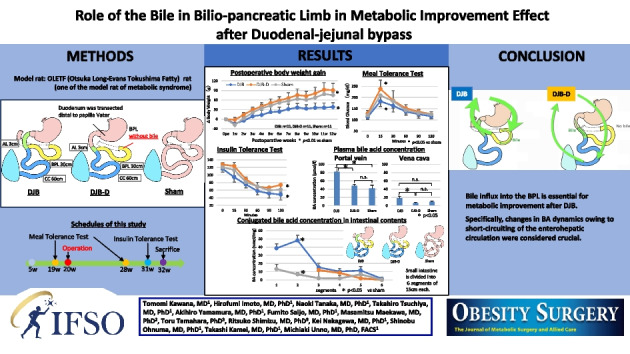

## Introduction

Bariatric surgery reduces weight as well as resolves metabolic diseases [1, 2], while reducing their severity. Bariatric surgery can be restrictive or malabsorptive. Clinically, restrictive procedures include laparoscopic sleeve gastrectomy (LSG) or adjustable gastric banding. Combined procedures with restrictive and malabsorptive components include laparoscopic Roux-en-Y gastric bypass or sleeve gastrectomy with duodenal-jejunal bypass (LSG/DJB) [3]. Other effects of bariatric surgery, apart from its weight reduction effect, improve glucose metabolism [4]. Bypass procedures are more effective in reducing the severity of diabetes [5, 6]. The mechanisms of metabolic improvement after bypass surgery are possibly related to bile acid (BA) dynamics, gastrointestinal hormones, and alterations in the gut microbiota [7–10]. However, the underlying mechanisms remain unclear.

**Fig. 1:**
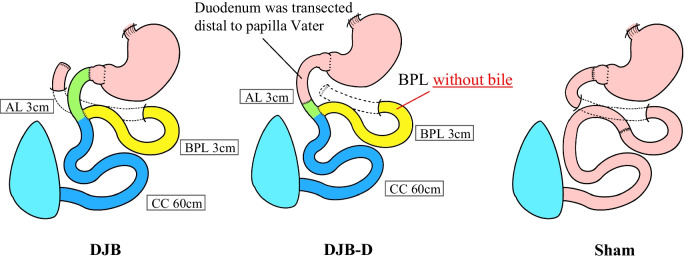
Schema of each procedure. **a** Schema of duodenal-jejunal bypass (DJB): alimentary limb (AL), biliopancreatic limb (BPL), and common channel (CC). **b** The length of each limb was as follows: DJB and DJB-D: AL 3 cm, BPL 30 cm, and CC 60 cm. In DJB-D, the duodenum is transected distal to the papilla of Vater. Sham: The duodenum and small intestine were transected and anastomosed 30 cm from the ligamentum

We have been studying the mechanisms of metabolic improvement following bariatric surgery using the duodenal-jejunal bypass (DJB) model in diabetic obese rats. DJB is a pure bypass model without gastrectomy, making it a suitable procedure for metabolic improvement by bypass surgery alone, excluding the restrictive component. The small intestine in DJB consists of three parts: alimentary limb (AL) for food passage, biliopancreatic limb (BPL) for digestive juices such as bile and pancreatic juice pass, and common channel (CC), where digestive juice and food mix (Fig. 1a). Initially, we reported a higher metabolic improvement effect in DJB with a longer BPL and elevated serum BA level [11]. Subsequently, we investigated the BA dynamics after DJB by altering BPL length. BPL length correlated with plasma BA level and the glycemic improvement effect, and early reabsorption of BAs in the BPL occurred after DJB [12, 13]. These results suggest that BPL is crucial in the mechanism of metabolic improvement after DJB and early reabsorption of BAs in BPL; thus, “shortening of the enterohepatic circulation” is considered the key mechanism. We hypothesized that if bile does not flow into the BPL, enterohepatic circulation would not be shortened and the metabolic improvement effect would decrease or be canceled. Thus, we established a novel DJB procedure that does not allow bile flow into the BPL by devising the position of duodenal-jejunal anastomosis of DJB.

On the other hand, gut microbiota is a topic in the recent treatment of obesity, and BAs interact with gut microbiota and regulate each other [14]. Gut microbiota is associated with glycolipid metabolism, the immune system, and gastrointestinal hormones [15]. Studies have observed changes in the gut microbiota between obese and non-obese individuals before and after bariatric surgery [15]. Therefore, we aimed to explore the relationship between gut microbiota alterations, metabolic improvement effects, and BA dynamics for each procedure to elucidate the mechanisms of metabolic improvement after DJB.

## Materials and Methods

### Animals

Otsuka Long-Evans Tokushima Fatty (OLETF) rats were used in this study. These rats lack cholecystokinin-A receptors and develop obesity and hyperglycemia with hyperinsulinemia after overeating [16–20]. They have been used as models for type 2 diabetes associated with obesity. Five-week-old male OLETF rats were procured from Japan SLC, Inc. (Shizuoka, Japan). All rats were single-housed under specific pathogen-free conditions and fed normal chow (Labo MR stock; 2.31 kcal/g Nosan Corporation, Yokohama, Japan), comprising 15.2%kcal fat, 32.6%kcal protein, and 52.2%kcal carbohydrates. The animal care and utilization committee of our institute approved all experiments (2021IDO-013–01).

### Operative Procedure

Operation was performed on 20-week-old diabetic rats after overnight fasting. The surgical procedure was performed according to our previous reports [11, 21]. OLETF rats with diabetes were categorized into three groups (*n* = 11 in each group): two DJB groups (DJB and DJB-D) and one sham group (Fig. 1b). In the DJB group, the duodenum was divided immediately distal to the pylorus, and the duodenal stump was closed. The jejunum was transected 30 cm distal to the ligament of Treitz, and the distal end of the jejunum was anastomosed end-to-end with the proximal end of the duodenum. The proximal end of the jejunum was anastomosed in an end-to-side fashion to the jejunum, 3 cm distal to the duodenal-jejunal anastomosis. DJB-D (distal) is a novel procedure in which bile does not flow into the BPL. Duodenal-jejunal anastomosis was performed distal to the papilla of Vater for preventing bile from flowing into the BPL. In the sham group, intestinal dissection was performed to account for the impact of surgical invasion, especially intestinal anastomosis, following our previous reports. The duodenum proximal to the pylorus and jejunum, 30 cm distal from the ligament of Treitz, was transected and anastomosed.

### Body Weight and Food Intake

Body weight and food intake were monitored weekly for 12 weeks postoperatively.

### Meal Tolerance Test and Insulin Tolerance Test

All examinations were performed after overnight fasting. The meal tolerance test (MTT) was performed 1 week preoperatively and 8 weeks postoperatively, respectively. Rats were administered a liquid mixed meal, Ensure HN (1.5 kcal/mL, 28 kcal% fat, 15 kcal% protein, and 57% carbohydrates, Abbott Japan Co., Chiba, Japan), through oral gavage at a dose of 1.2 g carbohydrates/5.8 mL/8.76 kcal/kg body weight. Blood was drawn from the tail vein at baseline and at 15, 30, 60, and 90 min after administration. Blood glucose levels were measured using LAB Gluco (Research and Innovation Japan). The insulin tolerance test (ITT) was performed 11 weeks postoperatively. Insulin was injected at a dose of 0.5 U/kg. Blood glucose levels were measured at baseline and at 15, 30, 60, and 90 min after injection.

### Blood, Tissue, and Intestinal Content Collection

Twelve weeks postoperatively, all rats were administered Ensure HN through oral gavage at the same dose as MTT after overnight fasting. All rats were euthanized 1 h after meal loading, and blood and intestinal contents were collected. Blood samples were drawn from the portal vein (PV) and inferior vena cava (IVC) and collected in ice-chilled tubes containing ethylenediaminetetraacetic acid. Plasma samples were obtained by centrifuging the blood (1300 g for 10 min at 4 ℃) and stored at − 80 ℃. Intestinal contents were stored at − 80 ℃.

### BA Analysis

The total BA levels in the PV and IVC were examined using a total BA test kit (Wako, Osaka, Japan). Each BA concentration was analyzed using liquid chromatography-mass spectrometry (LC–MS/MS). Plasma samples were diluted with methanol to prepare a sample solution, whereas intestinal contents were diluted after homogenization and used as a sample solution. Subsequently, 50 μL of the internal standard solution and 900 μL of water were added to 50 μL of the sample solution, followed by pretreatment using an OASIS WAX cartridge (Waters Co, Milford, MA, USA). The Nexera liquid chromatograph (Shimadzu Co., Ltd., Kyoto, Japan) and InertSustain C18 column (GL Science Co., Tokyo, Japan) were connected to a QTRAP 6500 tandem mass spectrometer (SCIEX, Framingham, MA, USA). Purified sample solutions were then analyzed using LC–MS/MS.

### Meta-analysis of 16S rRNA Gut Microbiota

DNA was extracted using the DNeasy PowerSoil Pro Kit (Qiagen GmbH, Hilden, Germany), and the V3–V4 region of the 16S rRNA genes was amplified, barcoded, and sequenced on the Illumina MiSeq platform following the manufacturer’s instructions. The primers for the V3–V4 region used for PCR amplification were sourced from Bioengineering Lab. Co., Ltd. (Sagamihara, Japan). Sequence data from the amplicons were analyzed using QIIME2 [22] version 2020–8. The first 20 bases of both sequences were trimmed for all paired reads to remove the primer sequences. The forward bases after position 280 and reverse bases after position 240 were truncated to remove low-quality sequence data, and potential amplicon sequencing errors were corrected using DADA2 [23] to generate an amplicon sequence variant (ASV) dataset. The resultant ASVs were aligned using MAFFT [24] and used to construct a phylogenetic tree using FastTree2 [25]. The α- and β-diversity metrics were estimated using processed ASV datasets. Each ASV was identified using a Naïve Bayes classifier trained on 16S rRNA gene sequences from Silva as the reference database (the Silva dataset) [26] and assigned at the species level. Principal coordinates and other statistical analyses were performed using Python and R scripts.

### Data Analysis

All data are presented as mean ± standard error of the mean. All statistical analyses were conducted using the JMP Pro 16.0.0 statistical software package (SAS International Inc., Cary, NC, USA). Statistical significance was set at a *p-*value of < 0.05. Repeated-measures analysis of variance was performed to compare changes over time among the three groups. The Tukey–Kramer or Steel–Dwass test was performed for other comparisons among the three groups.

## Results

### Weight Gain and Food Intake

**Fig. 2:**
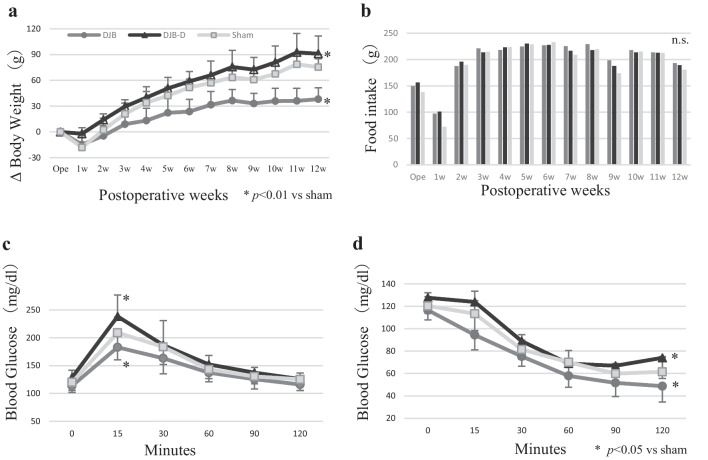
Effect of duodenal-jejunal bypass. **a** Postoperative body weight. **b.** Postoperative food intake **c.** Blood glucose levels in the meal tolerance test. **d** Blood glucose level in the insulin tolerance test. Data are presented as mean ± SEM; *n* = 11 rats per group (Dunnet test or Steel test after Levene test)

In the DJB group, weight gain was suppressed, whereas no such effect was observed in the DJB-D group (postoperative 12-week weight gain DJB group: 38.15 ± 4.0 g, DJB-D group: 91.06 ± 6.22 g, Sham group: 75.41 ± 2.92 g; Fig. 2a) In contrast, no differences in food intake were observed among the three groups (Fig. 2b).

### Glucose Metabolism

In MTT, the blood glucose levels remained considerably lower only in the DJB group, and the levels in the DJB-D group were similar to or slightly higher than those in the sham group (Fig. 2c).

In ITT, the blood glucose levels were considerably lower in the DJB group than in the other two groups, whereas those in the DJB-D group remained similar to or were slightly higher than those in the sham group (Fig. 2d).

### Serum Total BA Level

**Fig. 3:**
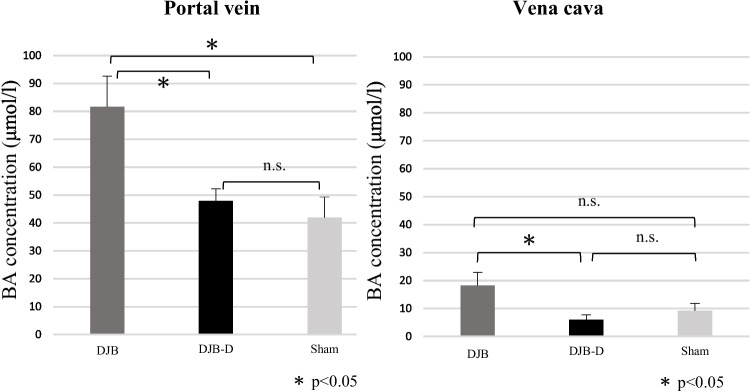
Plasma bile acid concentration. Data are presented as mean ± SEM; *n* = 11 rats per group (Dunnet or Steel test after Levene’s test)

The total BA levels in the PV and IVC were considerably higher in the DJB group than in the other two groups. However, no differences were observed in the total BA levels between the DJB-D and sham groups (Fig. 3).

### Conjugated BA Concentration in Intestinal Contents

**Fig. 4:**
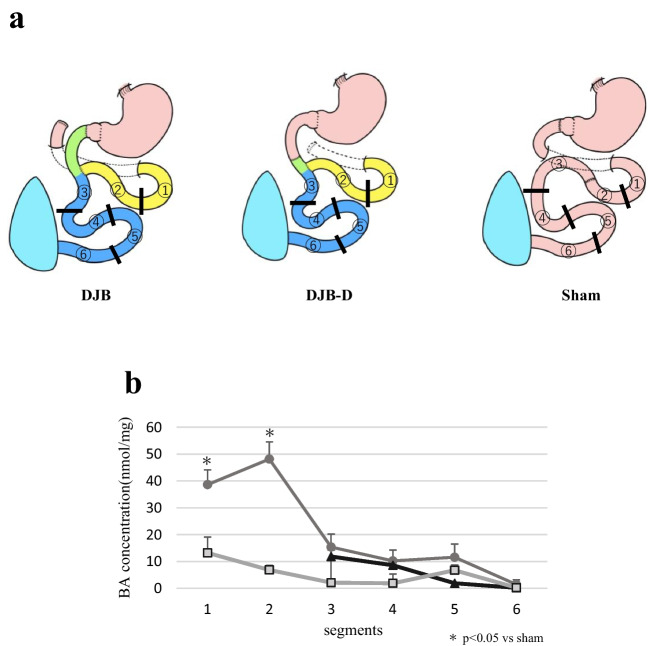
Analysis of bile acids in the intestine. **a** The small intestine was divided into six segments. **b** Conjugated bile acid concentration in intestinal contents. Data are presented as mean ± SEM; *n* = 11 rats per group; *p < 0.05 values in the sham group (Dunnet or Steel test after Levene test)

Figure 4a illustrates the division of the small intestine into six segments. BA concentration was measured only in the CC in the DJB-D group, where almost no BA was present in the BPL. In the DJB-D group, the conjugated BA concentration in the BPL was considerably higher than that in the sham group and rapidly decreased in the BPL toward the entrance of the CC (segment 3). There were no differences in the conjugated BA concentration in segments 3–6 among the three groups (Fig. 4b).

### Gut Microbiota

**Fig. 5:**
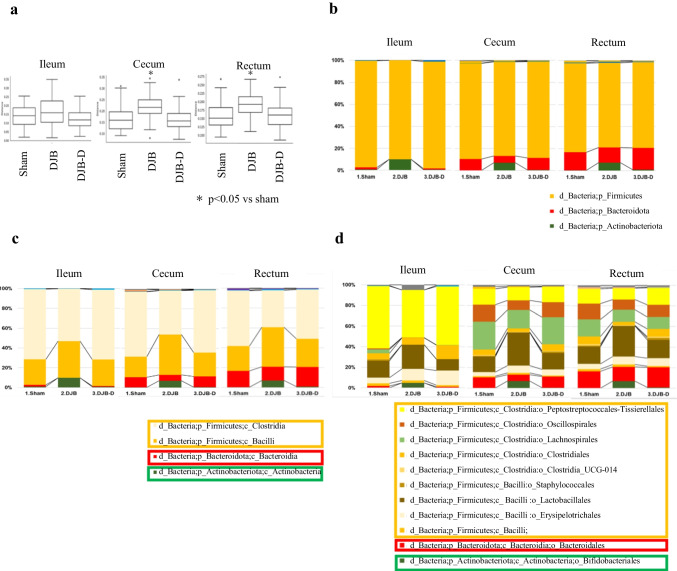
Gut microbiota analysis. **a** Principal component analysis of the gut microbiota. **b**, **c**, and **d** Gut microbiota composition ratio

Principal component analysis of the gut microbiota in the cecum and rectum along with meta-analysis of 16S rRNA gene sequences revealed differences in distribution between the DJB group and the other two groups. However, no difference was observed between the DJB-D and sham groups (Fig. 5a). The abundance of *Firmicutes* and *Bacteroidetes* decreased, whereas that of *Actinobacteria* increased in the DJB group. Furthermore, the abundance of the phylum *Firmicutes* decreased in the DJB group. However, the proportion of the *Lactobacillus* order increased only in the DJB group, whereas those of other orders remained low (Fig. 5b).

## Discussion

This study underscores the importance of the BPL, as well as the presence of bile and its role in the mechanism of metabolic improvement after DJB. We introduced a novel procedure, DJB-D, in which bile does not flow into the BPL to assess the significance of bile in the BPL and compared the DJB, DJB-D, and sham groups. Glucose metabolism improved and weight gain was suppressed in the DJB group alone. In contrast, this was not observed in the DJB-D group, as hypothesized. Therefore, bile in the BPL is deemed essential for metabolic improvement after DJB. These results confirm our previous findings that the presence of bile in the BPL enhances BA reabsorption, indicating that changes in BA dynamics owing to the “shortening of the enterohepatic circulation” are crucial in the mechanism of metabolic improvement after DJB. This phenomenon “shortening of the enterohepatic circulation in BPL” was first observed by our institution. In this study, we explored these findings and established and examined a novel procedure, i.e., DJB-D. Therefore, these findings are novel.

BAs are pivotal for regulating energy expenditure [27, 28]. They affect various organs, including the brown cells, liver, pancreas, and muscles; increase gluconeogenesis; promote insulin secretion; and decrease appetite [29]. BAs synthesized in hepatocytes are conjugated with glycine and taurine and secreted into the duodenum as bile [30]. Then, the gut microbiota converts BAs into secondary BAs, 95% of which are reabsorbed in the terminal ileum. The reabsorbed BA circulates through the PV to the liver and is secreted back into the bile—“enterohepatic circulation” [30]. This continuous recycling maintains a BA pool of 2–4 g, occurs 6–9 times daily, and regulates BA production [31, 32]. BA dynamics change after bariatric and metabolic surgery, resulting in elevated blood BA concentrations that contribute to metabolic improvement [33]. In particular, increased BA concentration is reportedly associated with improved glucose and lipid metabolism, which are mediated by the farnesoid X receptor (FXR) and transmembrane G-protein coupled receptor 5 (TGR5) [34]. FXR plays a role in regulating glucose and lipid metabolism, and energy expenditure [35]. TGR5 activation on the L-cells in the small intestine induces the release of glucagon-like peptide-1 and is associated with improving glucose metabolism [36]. In this study, given these changes in BA dynamics in the DJB-D group, BA reabsorption in the BPL—shortening of enterohepatic circulation—did not occur owing to the absence of bile in the BPL, resulting in no postoperative metabolic improvement.

Furthermore, BA composition is influenced by gut microbiota, which dehydrates amino acids in BAs and produces secondary BAs through the dehydroxylation of hydroxyl groups. This process attenuates FXR signaling in the liver [37]. Additionally, BAs function and influence gut microbiota composition through their bactericidal, bacteriostatic, and germination-inducing effects. This interplay between BAs and the gut microbiota regulates their functions [14, 38].

Generally, patients with obesity exhibit a reported decrease in the abundance of the *Bacteroidota* phylum and an increase in the abundance of the *Firmicutes* phylum within their gut microbiota. After bariatric surgery, these trends are reversed [15, 39–44]. Additionally, the abundance of the phylum *Actinobacteria* increases [42, 44–46]. In this study, gut microbiota composition changed only in the DJB group, parallel to the changes in BA dynamics, and may also have affected the metabolic improvement. In detail, the abundance of the phyla *Firmicutes* and *Actinobacteria* decreased and increased, respectively, in the DJB group. Furthermore, the abundance of numerous orders within the *Firmicutes* phylum decreased, whereas that of the *Lactobacillales* order increased. *Lactobacillales* are linked to glucose metabolism homeostasis [47], suggesting their passive involvement in metabolic improvement after DJB. The *Actinobacteria* phylum, which mainly includes the *Bifidobacterium* genus, can decompose lactose and other substances to produce lactic and acetic acids, lowering the pH in the intestine and regulating its environment [38, 48]. However, the relationship between this effect and metabolic improvement remains unclear. These findings are consistent with those of a previous report on changes in gut microbiota after metabolic surgery and support their relationship with metabolic improvements.

Clinically, in our institution, we have obtained good results in improving glucose metabolism by lengthening the BPL to 150 cm in LSG/DJB. Furthermore, applying this mechanism may lead to future studies on the appropriate limb length in metabolic surgery with intestinal bypass, the utility of a longer BPL in Roux-en-Y reconstruction of gastric cancer patients with diabetes, and the possibility of improving metabolism without surgery by inducing similar changes after DJB.

Various factors are intricately related to the mechanism of metabolic improvement after DJB; nonetheless, we focused on the significance of bile in BPL and the alteration of gut microbiota in this study. These results support that bile in BPL is essential for metabolic improvement after DJB and enterohepatic circulation shortens in the BPL. Simultaneously, changes in the gut microbiota are related to the mechanism of metabolic improvement after DJB.

## Limitation

Our study has some limitations. We used rats as models in this study, although BA fractions vary greatly among animal species. In humans, bile secreted from the liver is stored in the gall bladder and is concentrated; however, rats do not have a gallbladder. Therefore, whether the postoperative changes in BA kinetics in rats apply to humans is unknown. Further studies are required to determine the mechanism of BA reabsorption in BPL in a clinical setting involving humans.

Another limitation of this procedure is the difficulty in eliminating the influence of pancreatic juice since bile is present with pancreatic juice in DJB and DJB-D. Separation of bile and pancreatic juice is particularly difficult in a rat model without a gallbladder and remains a challenge for the future.

## Conclusion

Bile influx into the BPL is essential for metabolic improvement after DJB. Specifically, changes in BA dynamics owing to short-circuiting of enterohepatic circulation are considered crucial. Furthermore, the gut microbiota was simultaneously altered with changes in BA dynamics, suggesting its involvement in metabolic improvement after DJB. We hope this study elucidates the mechanisms of metabolic improvement in metabolic surgery.

## Symbols Used

β: Beta
α: Alpha
≤: Less than or equal to
±: Plus-minus
%: Percentage
